# Temporal Association of Herpes Simplex Virus ICP4 with Cellular Complexes Functioning at Multiple Steps in PolII Transcription

**DOI:** 10.1371/journal.pone.0078242

**Published:** 2013-10-11

**Authors:** Lauren M. Wagner, Neal A. DeLuca

**Affiliations:** Department of Microbiology and Molecular Genetics, University of Pittsburgh School of Medicine, Pittsburgh, Pennsylvania, United States of America; Dartmouth Medical School, United States of America

## Abstract

The herpes simplex virus type 1 (HSV-1) immediate early protein, ICP4, participates in the regulation of viral gene expression by both activating and repressing RNA polII transcription. We used affinity purification of ICP4 expressed in infected cells followed by mass spectrometry and western blot analysis to determine the composition of cellular complexes associated with ICP4 throughout infection. ICP4 was associated with TFIID complexes containing a distinct set of TAFs. These complexes were most abundant early, but were detected throughout infection, whereas Mediator was found in ICP4 containing complexes later in infection, indicating a temporal pattern for the utilization of these complexes for the transcription of the viral genome. The form of Mediator copurifying with ICP4 was enriched for the kinase domain and also lacked the activator-specific component, Med26, suggesting that Mediator-ICP4 interactions may be involved in repression of viral transcription. The N-terminal 774 amino acids of ICP4, which retains partial function, were sufficient to form complexes with TFIID and Mediator, although these interactions were not as strong as with full-length ICP4. Additionally, components involved in transcription elongation, chromatin remodeling, and mRNA processing were isolated with ICP4. Together our data indicate that ICP4 plays a more integrated role in mediating HSV transcription, possibly affecting multiple steps in transcription and gene expression.

## Introduction

 The genome of Herpes Simplex Virus Type 1 (HSV-1) is transcribed by RNA polymerase II (RNA polII) [1]. Transcription of the viral genome follows a coordinately regulated cascade in which roughly three temporal classes of genes exist [2,3]. Immediate early (IE) genes are transcribed in the absence of prior protein synthesis, largely due to VP16 present in the infecting virion subsequently acting on the promoters of IE genes to activate their transcription [4-6]. Functional IE proteins are required for the transcription of early (E) genes [3], the products of which are involved in viral DNA synthesis. The syntheses of IE and E proteins along with viral DNA replication are prerequisites for efficient late (L) transcription, the protein products of which mostly comprise the virion structure or are required for its assembly.

 The IE protein, Infected Cell Polypeptide 4 (ICP4), is an activator and repressor of transcription. ICP4 binds to a specific DNA sequence [7-9]. Properly positioned binding sites in several viral genes mediate transcriptional repression by ICP4 (reviewed in 10), although this mechanism appears to be distinct from the attenuation of most IE and E transcription later in infection. ICP4 is absolutely required for the transition from IE transcription to later viral gene transcription [11-14]. The DNA binding function of ICP4 is necessary [15,16] but not sufficient to activate viral gene expression [16,17]. ICP4 activates transcription by the recruitment of a form of TFIID to promoters, suggesting a relatively promiscuous mechanism for activation [17-19]. 

 While interactions between ICP4 and components of TFIID and Mediator have been demonstrated [17,18,20], the forms of TFIID and Mediator that associate with ICP4 are currently not known. These cellular complexes can exist in different forms to affect the transcription of different sets of genes. Additionally, ICP4 exists in cells as a 350 kD dimer, having hydrodynamic properties that suggest an elongated conformation [21]. Therefore, given its size and complex structure, it is likely that ICP4 interacts with a greater set of proteins to regulate viral gene expression. Lastly, the temporal association of ICP4 with cellular transcription factors as infection proceeds might also contribute to its role in regulation. Therefore, the goals of these studies were to i) determine the composition of TFIID and Mediator associated with ICP4 during infection, ii) whether the interactions between ICP4, TFIID, and Mediator change over the course of infection, iii) determine the genetic requirements of ICP4 for these interactions, and iv) isolate novel ICP4 containing complexes. To accomplish these goals, we generated wild-type (wt) and mutant ICP4 expressing viruses containing tandem affinity purification tags at the amino terminus of ICP4. This allowed us to isolate complexes interacting with ICP4 during viral infection, where ICP4 and all the other viral proteins are produced in the amounts normally synthesized during infection. 

## Materials and Methods

### Cells and Viruses

Vero cells were maintained as suggested by ATCC. E5 cells express complementing levels of ICP4, and have been described previously [22]. The viruses, wild type strain KOS and n208 [23], have been previously described. TAP-KOS and TAP-n208 were generated for this study by marker transfer with the TAP-pK1-2 and TAP-pn7 plasmids (described below), and their genotypes were confirmed by Southern blot and sequencing. 

### Recombinant Plasmids

TAP-ICP4 plasmids were constructed such that the TAP sequence would be in frame and 5’ to the ICP4 translation start site. The 77 amino acid TAP tag consists of a tandem arrangement of the calmodulin binding protein and the streptavidin binding protein from the plasmid pNTAP-B (Statagene). wtICP4 and n208 plasmids, pk1-2 and pn7 were utilized to construct the TAP-pk1-2 and TAP-pn7, respectively, by the RecET recombination system, as described previously [24,25]. The primers used in the initial recombination step to insert the TAP tag contained homology to ICP4 -60 to -20 and -7 to +31, relative to the ICP4 translation start site. 

### Streptavidin Affinity Purification

2 x 10^8^ Vero cells were infected at a multiplicity of 10 pfu/cell with KOS, TAP-wtICP4, n208, or TAP-n208 for 3, 6, or 12 hours at 37°C prior to harvesting. Cells were rinsed twice with TBS containing 0.1 mM *N-p-*tosyl-L-lysine chloromethyl ketone (TLCK) and scraped into 50 mL TBS + 0.1 mM TLCK. Cell pellets were collected by centrifugation at 5 K for 5 min in the Sorvell RC5 centrifuge. Nuclei were collected and proteins were extracted in 0.4 M KCl as described previously [20]. Nuclear extracts were diluted in half in 2 X low salt streptavidin binding buffer (2 X LS SBB) (20mM Hepes KOH pH 7.9, 20 mM KCl, 4 mM EDTA, 0.2 % NP40), treated with 300 U benzonase nuclease (Novagen) and incubated with 500 uL streptavidin conjugated agarose beads (Pierce) overnight at 4°C with end over end rotation. Beads were washed 4 times in 10 mL of streptavidin binding buffer (SBB) (20 mM Hepes KOH pH 7.9, 200 mM KCl, 2 mM EDTA, 0.1% NP40). Samples were eluted from the beads using 500 uL streptavidin elution buffer (SBB + 2 mM Biotin). Two elutions, each 30 min, and one elution, overnight, were combined and either concentrated in 7 mL/9 kDa iCon concentrator (Pierce) and used for western blot analyses, or sent to MS Bioworks (Ann Arbor, MI) for TCA precipitation, in gel digestion, and LC-MS/MS analysis. 

### LC-MS/MS Analysis

Samples were sent to MS Bioworks (Ann Arbor, MI) for processing. Breifly, protein samples were precipitated with TCA and separated on a 10% Bis-Tris Novex mini-gel using a MES based system, stained with Coomassie stain, and the gel was divided into 10 equal sized slices. Gel fragments were processed using the ProGest (DigiLab) robot. The gel slices were washed with 25 mM ammonium bicarbonate followed by acetonitrile. The samples were reduced with 10 mM DTT at 60°C followed by alkylation with 50 mM iodoacetamide at room temperature. The protein samples were digested with trypsin (Promega) for 4 hours at 37°C which was quenched with formic acid. The supernatant was then loaded onto a Waters NanoAcquity HPLC system interfaced to an Orbitrap Velos Pro (Thermo-Fisher). Data was analyzed against the *Rhesus macaque* and HHV-1 proteomes using the MASCOT program and validated using Scaffold software. 

### Electrophoresis, Silver Stain, and Western Blot

SDS polyacrylamide gel electrophoresis was carried out essentially as previously described [26]. Silver stained gels were processed according to the manufacturers directions (Pierce). For western blotting, proteins were transferred to either polyvinylidine fluoride (PVDF; Amersham) for chemi-luminescent detection with ECL reagent (Amersham) or nitrocellulose membranes for infrared detection by fluorescence using the LI-COR Odyssey infrared imager according to the manufacturers instructions. The antibodies used to probe the membranes were; N15, polyclonal rabbit serum, for ICP4 (1:500), Trap220/Med 1 (sc-8998-X), Med6 (sc-9434), Med 4 (NBP1-84977; Novus Biologicals), Med23 (550429; BD Bio), CDK8 (sc-1521), Med12 (NB100-2357; Novus Biologicals), Med 13 ((NB100-60642; Novus Biologicals), Med 26 (sc-166614), TAF1 (sc735), TBP (233-R Covance), p62 (sc292-X), p80/XPB (sc-20696-x), CPSF2 (sc-165983), CPSF7 (NB100-61600; Novus Biologicals), Brahma (sc-6450), CHD3 (ABE86; Milipore), RUVBL1 (06-1299; Milipore), RUVBL2 (06-1300; Milipore) at the manufacturers recommended dilutions.

## Results

**Figure 1:**
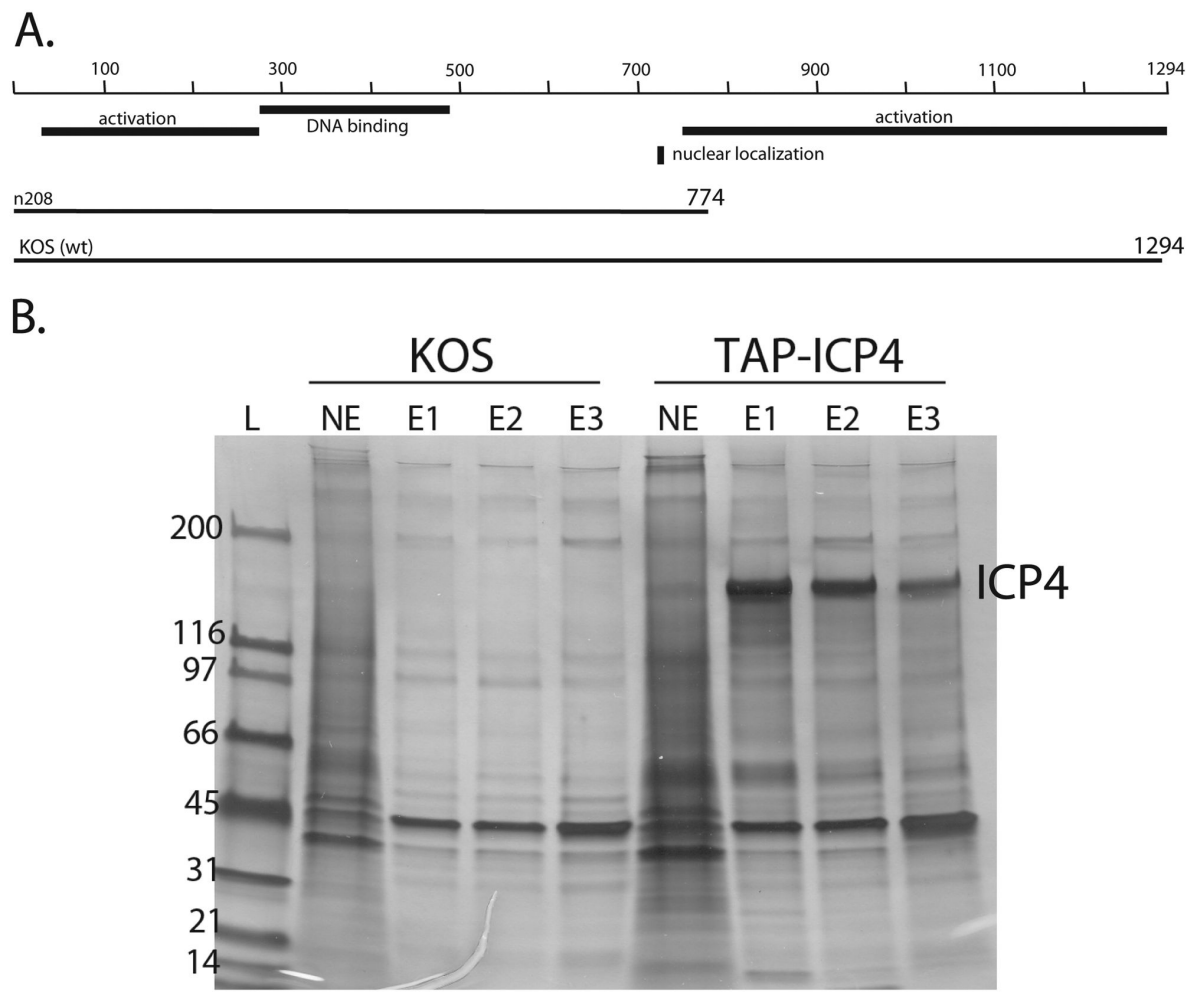
ICP4 molecules used in this study. (**A**) An affinity purification tag (TAP) was appended to amino acid 1 of the 1294 and 774 amino acid wild-type (wt) KOS and n208 molecules in the HSV genome. Also shown are regions involved in DNA binding, transactivation and nuclear localization. (**B**) Silver stain analysis of TAP-wtICP4 containing complexes. Samples were purified at 6 hpi using either wtICP4 (KOS) or TAP-wtICP4 (TAP-ICP4) expressing viruses. Nuclear extracts (NE) were loaded onto a streptavidin column and the affinity purified ICP4 was eluted from the streptavidin beads in three separate elutions (E1, E2, and E3). The resulting proteins were separated via SDS-PAGE and visualized by silver stain. Molecular weight standards are shown to the left (L).

To better understand the mechanisms underlying viral transcription, the complexity of ICP4 mediated protein interactions during HSV-1 infection were investigated. A Tandem Affinity Purification (TAP) tag was inserted at the amino terminus of the wtICP4 (HSV-1, strain KOS) and a C-terminal truncation mutant, n208 (Figure 1A). The viral mutant n208 lacks the C-terminal 520 amino acids of ICP4, yet still partially functions as an activator [17]. However, it is defective for L gene expression, and viral yield is reduced 100-fold [23]. The n208 protein is also defective relative to wt ICP4 for activation of a late promoter in reconstituted in vitro transcription reactions [17]. To examine the temporal characteristics of ICP4 mediated interactions, KOS and TAP-wtICP4 infected samples were collected at 3, 6, and 12 hours post infection (hpi) as these time points traverse the entire cascade of HSV gene expression. n208 and TAP-n208 samples were collected at 6h post infection. Nuclear extracts were then prepared and ICP4 was affinity purified as specified in the Materials and Methods. 

**Figure 2:**
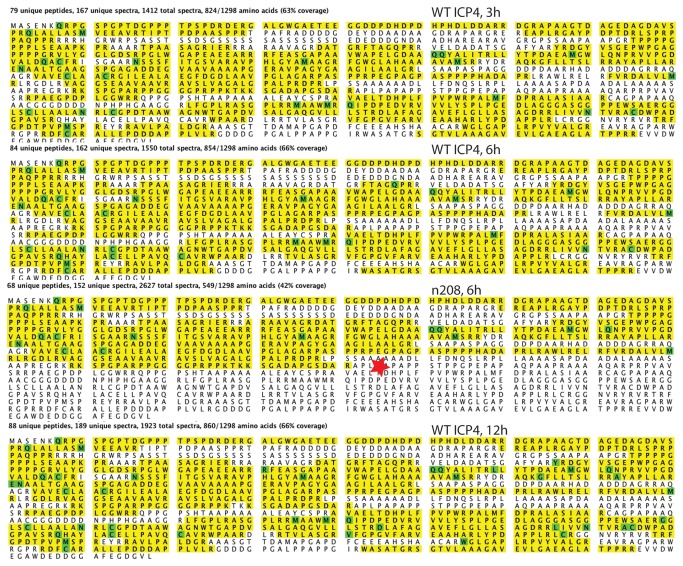
LC-MS/MS peptide coverage and spectral counts of ICP4 affinity purified form infected cells. The peptide coverage of ICP4 for the 3, 6, and 12 h infections and the n208 infection is shown highlighted in yellow relative to the amino acid sequence of ICP4. The green highlighted amino acids are those recognized as modified. The red star is location of the engineer translational stop codon in n208.

The composition of affinity-purified material was assessed by SDS-PAGE and silver staining (shown for KOS and TAP-wtICP4, Figure 1B). The biotin-eluted material from both samples was precipitated and the protein composition was identified by LC-MS/MS analysis. Untagged wtICP4 and n208 infections were analyzed in parallel to their TAP-tagged counterparts to eliminate nonspecific background. Figure 2 shows the coverage and numbers of spectral counts of TAP-ICP4 in the KOS and n208 samples. There were 1412, 1550, and 1923 spectral counts for ICP4 in the 3, 6 , and 12 h samples, respectively. This is consistent with the relative amounts of ICP4 in infected cells at these times. The amino acid coverage was very similar at all three time points (63-66%). The amino acid coverage for n208 is 42% relative to the wt ICP4 protein, but is similarly 64% relative to the amino acids expressed in n208. There were no peptides detected in the n208 sample corresponding to the region C-terminal to the engineered stop codon (Figure 2, star). These results are also summarized in table 1. 

**Table 1:** TFIID and Mediator components copurifying with ICP4.

			wtICP4 3hpi			wtICP4 6hpi			n208 6hpi			wtICP4 12hpi		
		M.W.^a^	SpC^b^	b/a	%C	SpC	b/a	%C	SpC	b/a	%C	SpC	b/a	%C
HSV	ICP4	133	1412	10.6	63	1550	11.6	66	NA	NA	NA	1923	14.5	66
	n208	78	NA	NA	NA	NA	NA	NA	2627	33.7	71	NA	NA	NA
TFIID	TAF1	209	135	0.65	38	100	0.48	28	25	0.12	14	51	0.24	21
	TAF2	137	91	0.66	45	66	0.48	36	91	0.66	45	40	0.29	26
	TAF3	87	25	0.29	25	8	0.09	8.8	6	0.07	9.7	15	0.17	17
	TAF4	68	68	1.00	58	42	0.62	50	37	0.54	56	33	0.49	49
	TAF4B	69	2	0.03	2.9	2	0.03	3.6	0	0.00	0	0	0.00	0
	TAF5	87	131	1.51	69	66	0.76	55	24	0.28	21	76	0.87	55
	TAF6	72	95	1.32	65	76	1.06	59	10	0.14	14	51	0.71	58
	TAF7	40	15	0.38	32	7	0.18	21	3	0.08	11	6	0.15	17
	TAF8	33	23	0.70	54	9	0.27	22	11	0.33	39	7	0.21	39
	TAF9	28	7	0.25	65	2	0.07	31	6	0.21	64	0	0.00	0
	TAF9B	26	47	1.81	78	24	0.92	52	37	1.42	74	17	0.65	47
	TAF10	22	9	0.41	22	8	0.36	25	7	0.32	22	6	0.27	22
	TAF11	23	11	0.48	33	4	0.17	5.2	2	0.09	9.5	0	0.00	0
	TAF12	18	24	1.33	32	14	0.78	27	14	0.78	27	19	1.06	30
	TBP	36	24	0.67	22	16	0.44	28	4	0.11	3.7	10	0.28	12
Mediator (head)	MED6	28	0	0.00	0	9	0.32	22	10	0.36	38	3	0.11	18
	MED17	72	9	0.13	15	16	0.22	29	0	0.00	0	18	0.25	23
	MED18	23	0	0.00	0	7	0.30	25	7	0.30	14	10	0.43	25
	MED20	23	7	0.30	19	14	0.61	28	18	0.78	30	14	0.61	39
Mediator (middle)	MED1	161	0	0.00	0	23	0.14	17	9	0.06	6.3	8	0.05	3.2
	MED4	30	0	0.00	0	8	0.27	30	9	0.30	25	4	0.13	16
	MED7	27	0	0.00	0	8	0.30	25	4	0.15	22	0	0.00	0
	MED31	15	0	0.00	0	6	0.40	32	2	0.13	20	7	0.47	34
Mediator (tail)	MED14	161	2	0.01	2.1	30	0.19	18	40	0.25	30	25	0.16	12
	MED15	76	0	0.00	0	8	0.11	10	0	0.00	0	5	0.07	7.7
	MED16	96	3	0.03	3.5	22	0.23	19	2	0.02	4.1	18	0.19	19
	MED23	156	2	0.01	1.4	41	0.26	26	33	0.21	20	33	0.21	21
	MED25	83	0	0.00	0	10	0.12	11	0	0.00	0	8	0.10	7.4
	MED27	31	0	0.00	0	8	0.26	29	7	0.23	29	5	0.16	23
Mediator (kinase)	MED12	244	11	0.05	4.7	58	0.24	20	14	0.06	6.9	31	0.13	15
	MED13	240	0	0.00	0	23	0.10	9.7	3	0.01	1.2	7	0.03	3.9
	M13L	242	0	0.00	0	5	0.02	2.3	0	0.00	0	5	0.02	1.7
	CYC C	35	0	0.00	0	9	0.26	27	5	0.14	18	5	0.14	12
	CDK8	53	0	0.00	0	5	0.09	6.3	0	0.00	0	2	0.04	4.5

a M.W. Molecular weight predicted from the primary amino acid sequence.

b SpC Spectral counts detected.

%C Percent coverage of the primary sequence with detected peptides.

Search results for ICP4 interacting partners were filtered to contain proteins that were at least 4x more abundant in the TAP-tagged samples than in the untagged samples and had a minimum of 5 spectral counts. Based on these criteria, 82, 141, 215, and 188 proteins were identified in the TAP-wtICP4 3 hpi, 6 hpi, 12 hpi, and TAP-n208 6 hpi samples, respectively. 

### TFIID- and Mediator-ICP4 Complexes

**Figure 3:**
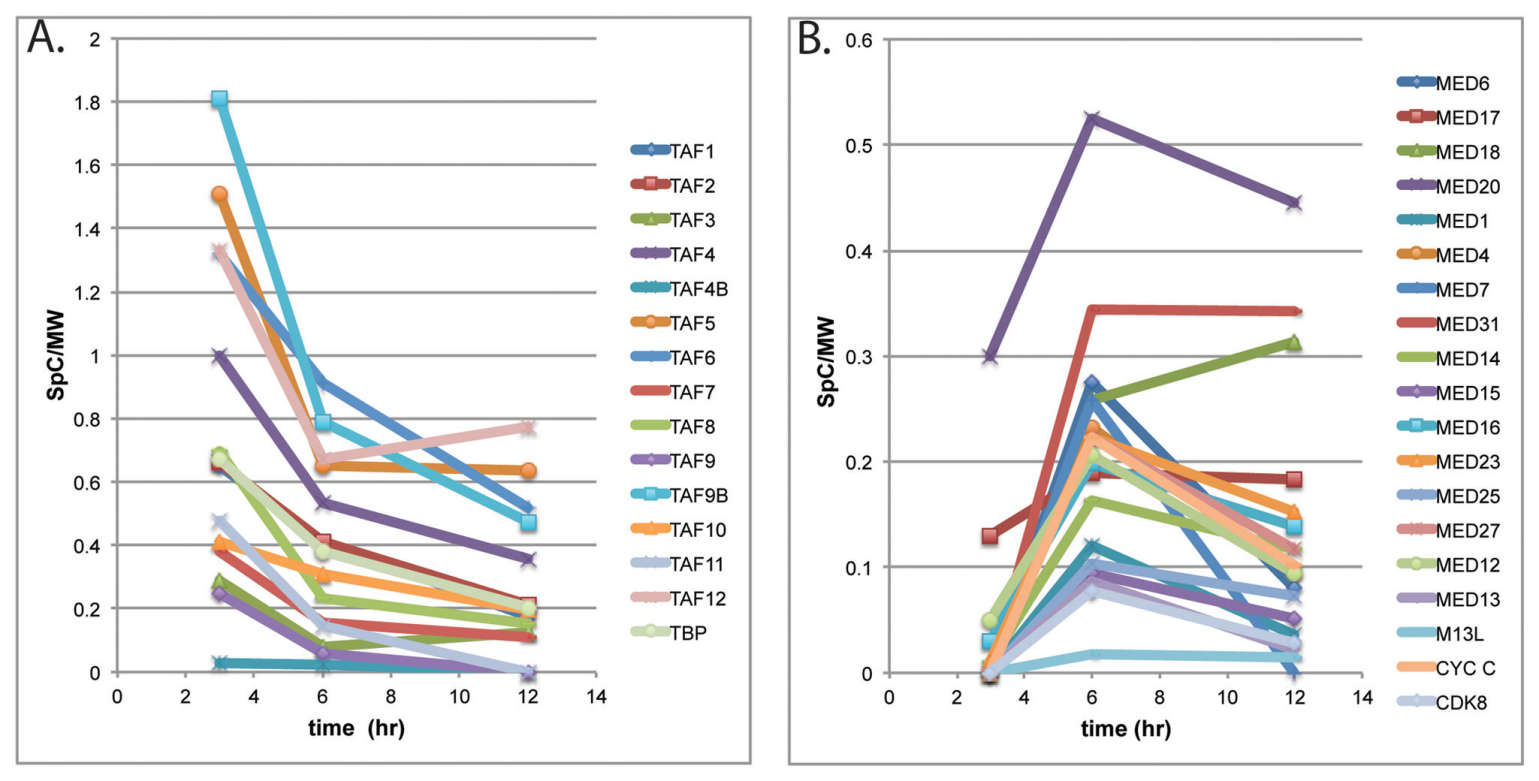
Normalized spectral counts for TFIID and Mediator components copurifying with ICP4 as a function of time post infection. **A**. The normalized spectral counts/MW for each component of TFIID detected by LC- MS/MS are plotted as a function of time post infection. **B**. The normalized spectral counts/MW for each component of Mediator detected by LC- MS/MS are plotted as a function of time post infection.

 All the components of TFIID and approximately 60% of the components of Mediator were identified in complex with TAP-wtICP4 by tandem LC-MS/MS. The number of spectral counts and the percent coverage for the detected components of TFIID and Mediator are listed in Table 1. While both of these parameters reflect the relative amounts of the proteins present, table 1 also lists the number of spectral counts divided by the molecular weight of the protein. This is to partially account for the fact that larger proteins will generate more spectral counts by virtue of their increased size. This parameter, the number of spectral counts/molecular weight, was used as a measure of the relative amounts of a given protein, and is plotted for the TFIID and Mediator components found in the samples from the wt virus infections in Figure 3. An important observation in this analysis was that there were no spectral accounts for TFIID or Mediator components recovered in any of the experiments in the parallel control lacking the TAP tag (see Figure 1B, KOS lanes, for example). 

More spectral counts of TFIID components were isolated with affinity purified ICP4 than any other cellular proteins. In fact the number of spectral counts for some of the TFIID components exceeded 10% of the number of spectral counts of ICP4 when normalized for MW (Table 1). The components of TFIID were found in complex with ICP4 as early as 3 hpi and these interactions were retained throughout infection. However, the relative abundances of the TAFs decreased over the duration of infection (Table 1, Figure 3A). TAFs 4,5,6, 9B and 12 were present in the greatest amounts in TAP-wtICP4 samples, with their normalized spectral counts being nearly double any other component of TFIID (Figure 3A). Interestingly, these TAFs are thought to be the core components of TFIID [27]. Additionally, TAF4 and TAF9B where detected relative to TAF4B and TAF9, respectively. In addition to the core TAFs, TBP and TAFs 1, 2, 3, 7, 8, 10 and 11 were detected. To our knowledge no viral activator has been previously shown to interact with TFIID complexes containing such a comprehensive collection of TAFs.

**Figure 4:**
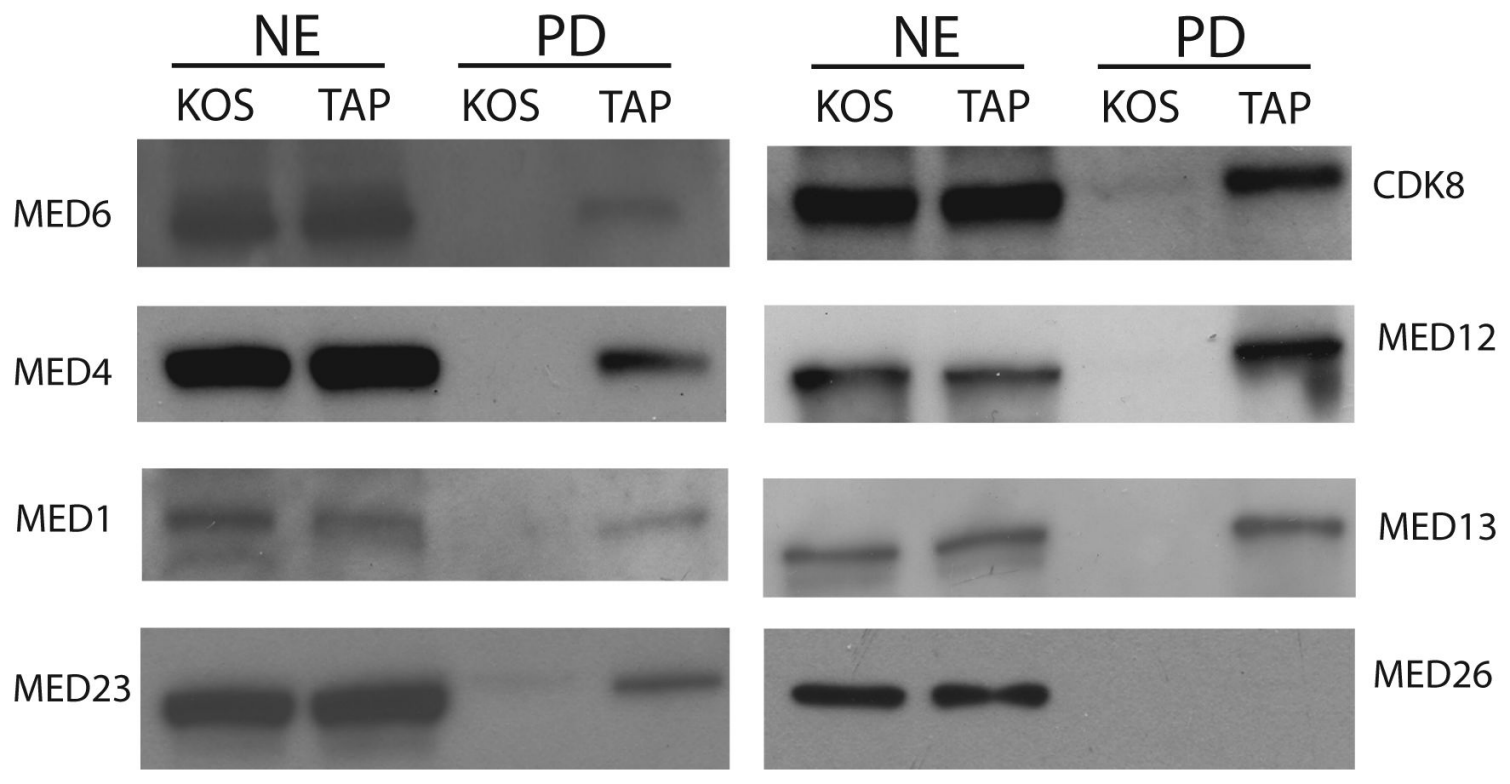
Components from all modules of Mediator are found in TAP-wtICP4 samples. Western blot analysis of nuclear extracts (NE) or affinity purified (PD) samples using either wtICP4 (KOS) or TAP-wtICP4 (TAP) expressing viruses at 6 hpi. Results of the Western blot analysis for components of the head (Med6), middle (Med4 and Med1), tail (Med23), kinase (cdk8, Med12, and Med13), and the Med26 protein are shown.

Mediator is a coactivator composed of approximately 30 subunits, divided into 4 modules, the head, middle, tail and kinase. Eighteen of the Meditator subunits distributed across the four modules were isolated with TAP-wtICP4 (Table 1). Western blot analyses were conducted on independently generated TAP and control samples. MED 6 of the head, MEDs 1 and 4 of the middle, MED 23 of the tail, and MEDs 12, 13, and CDK 8 of the kinase modules were all present in the TAP samples and absent in the parallel untagged control sample confirming the LC-MS/MS results (Figure 4). Of note, Med 26, which serves as a docking site for elongation factors and is associated with a coactivator form of Mediator [28,29], was absent from all preparations (Figure 4, Table 1). Additionally, all four subunits of the kinase module, which is typically associated with a repressive function of Mediator [30,31], were identified by LC-MS/MS. 

The number of spectral counts of mediator components that copurified with ICP4 was considerably less than for TFIID components (Table 1, Figure 3). Also unlike with TFIID, only 5 subunits of Mediator were identified by LC-MS/MS at 3 hpi. While the normalized spectral counts for TFIID components decreased from 3 to 6 hpi the normalized spectral counts for all the detected components of Mediator in the same sample increased during this time (Table 1, Figure 3). This indicates while ICP4 is found in complexes with TFIID at 3 hpi, the associations between ICP4 and Mediator become more prevalent later in infection (6 h). The situation is more complex at 12 hpi. Here several components remained relatively abundant (Meds 20, 31, 18, and 17), while the remainder of the detected Med components decreased in abundance relative to the 6h sample. Three of these proteins are components of the head module, suggesting the specific retention of this part of the mediator in ICP4-containing complexes late in infection. This is further discussed below. 

**Figure 5:**
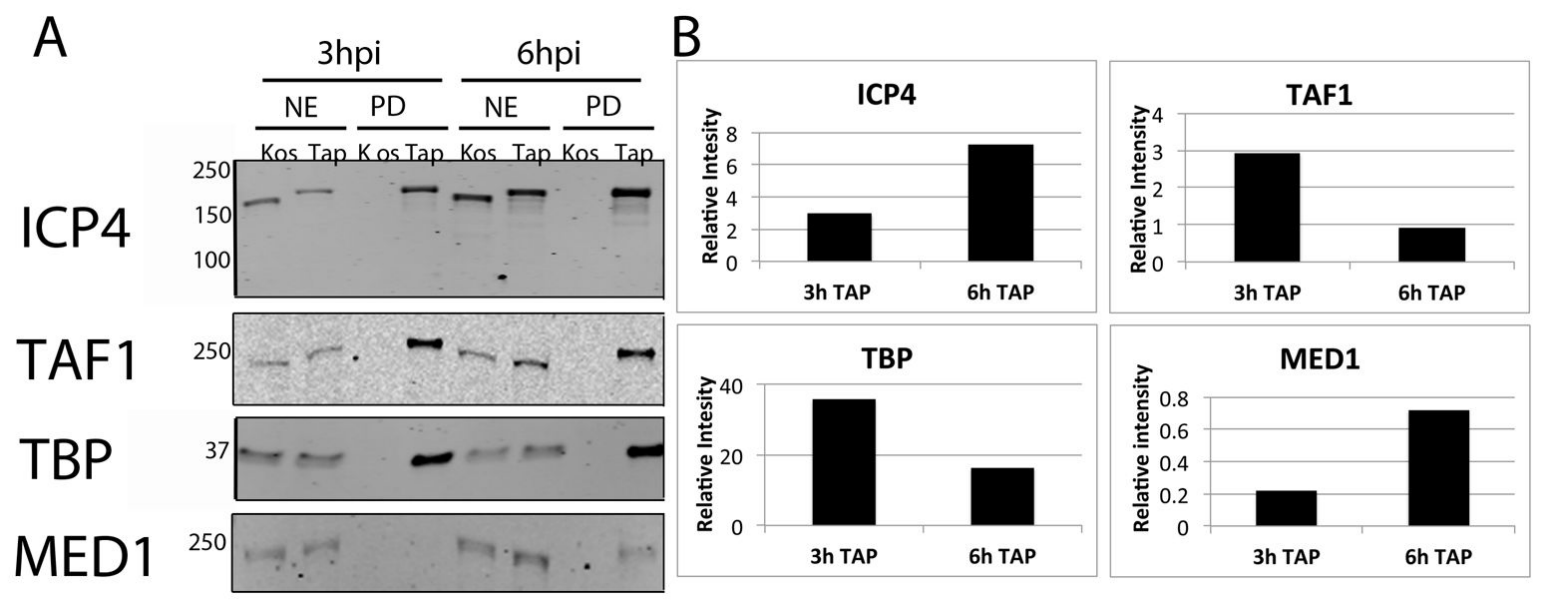
TFIID and Mediator display different temporal associations with ICP4. (**A**) Western blot analysis of nuclear extracts (NE) or affinity purified (PD) samples collected at either 3 hpi or 6 hpi using either wtICP4 (KOS) or TAP-wtICP4 (TAP) expressing viruses. Antibodies directed against ICP4, TAF1 and TBP of TFIID, and Med1 of Mediator were used. (**B**) Quantification of the western blots in (A). Images were quantified using the LiCOR Odyssey infrared imager and normalized to the amount of ICP4 present within the sample.

To confirm the temporal differences observed in the isolation of TFIID and Mediator with ICP4, independent samples were subjected to Western blot analysis and the fluorescent intensities of the resulting bands were quantified using a Licor Odyssey infrared imager (Figure 5). Figure 5A is the image of the Western blot of TAP-wtICP4 samples collected at 3 and 6 hpi probed with antibodies to ICP4, TAF 1, TBP, and MED 1, while Figure 5B is a graphic representation of the protein quantification analyses, normalized to the amount of ICP4. There was a 2-3-fold reduction in levels of TAF 1 and TBP at 6 hpi compared to 3 hpi (Figure 5B). Conversely, there was a 3-fold increase in the amount of MED 1 present in samples collected at 6 hpi compared to 3 hpi (Figure 5B). Together these data are consistent with the LC-MS/MS analyses and indicate that TFIID associates with ICP4 before Mediator. 

To determine whether the C-terminus of ICP4 was required for interactions with transcription complexes, potentially accounting for the observed deficiencies in L gene expression, TAP-n208 was purified at 6 hpi and the co-purifying proteins were compared to those isolated with TAP-wtICP4 at 6 hpi. Notably, all 12 TAFs and TBP were isolated with TAP-n208, albeit some TAFs were less abundant when compared to those isolated with TAP-wtICP4 (Table 1). Of the core TAFs, TAFs 4 and 12 were present at similar levels in both the TAP-n208 and TAP-wtICP4 samples, while TAFs 5 and 6 were decreased and TAF 9 was increased in the TAP-n208 samples (Table 1). The decreased levels of some, but not all, core components of TFIID in TAP-n208 samples could be indicative of direct interactions between the N-terminus of ICP4 and certain core components of TFIID such as TAF 9B, however this remains to be experimentally determined. 

**Figure 6:**
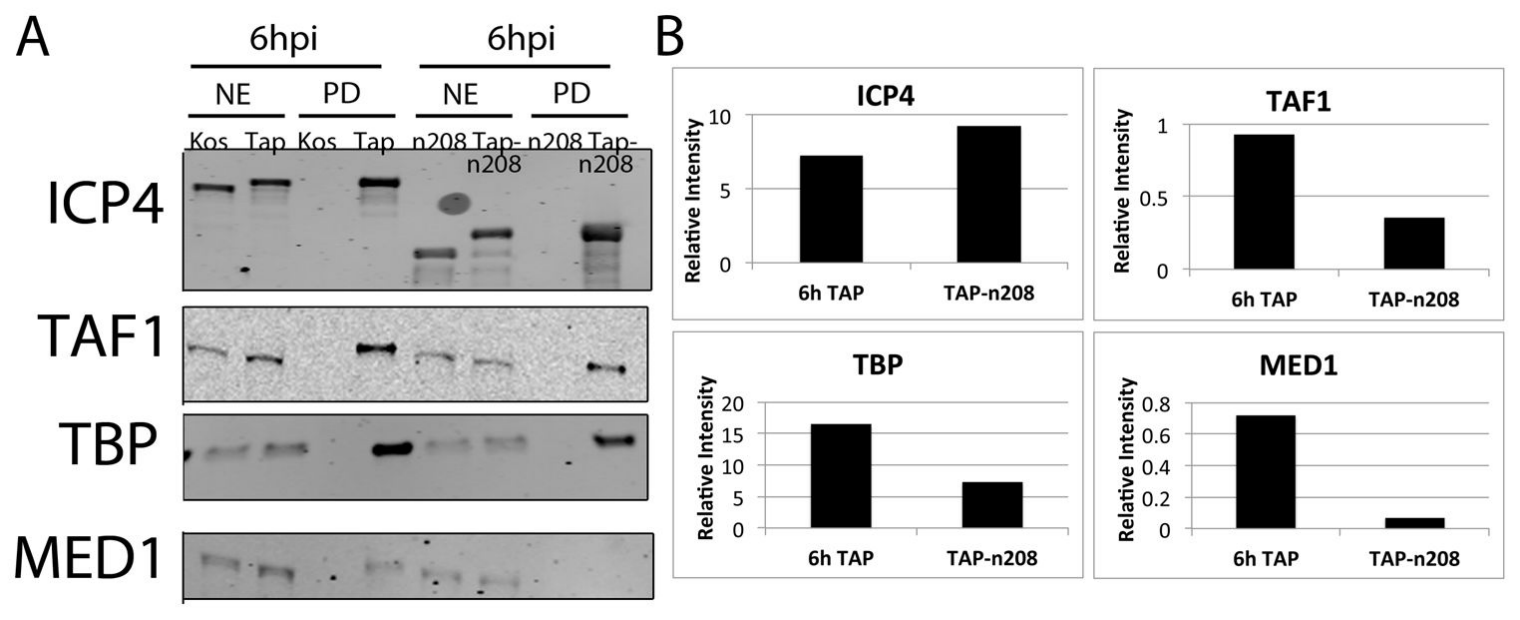
TAP-n208 associates with both TFIID and Mediator. (**A**) Western blot analysis of nuclear extracts (NE) or affinity purified (PD) samples collected at 6 hpi using either wtICP4 (KOS), TAP-wtICP4 (TAP), the C-terminal truncation mutant (n208) or a TAP-tagged C-terminal truncation mutant (TAP-n208) expressing viruses. Antibodies directed against ICP4, TAF1 and TBP of TFIID, and Med1 of Mediator were used. (**B**) Quantification of the western blots in (A). Images were quantified using the LiCOR Odyssey infrared imager and normalized to the amount of ICP4 present within the sample.

A comparison of proteins isolated from TAP-wtICP4 and TAP-n208 samples revealed that some components of Mediator were comparably isolated. These included MEDs 4, 6, 18, 20, 23, and 27 (Table 1). Conversely, other components of Mediator were less abundant in TAP-n208 purified samples. These include MEDs 1, 12, 13, 15, 16, 17, 25, cyclin C, and cyclin dependent kinase (CDK) 8. The existence of Mediator components in TAP-n208 samples suggests that the C-terminus of ICP4 is not necessary for interaction with Mediator. Interestingly, many of the Mediator components that were reduced in abundance in the TAP-n208 sample (7 out of 9) belong to the tail and kinase region of Mediator, perhaps suggesting an interaction between the N-terminal transactivation domain of ICP4 and a component of the head or middle regions of Mediator. To validate these results, Western blot and protein quantification analyses were performed on independently isolated TAP-wtICP4 and TAP-n208 samples collected at 6 hpi using antibodies to ICP4, TAF 1, TBP, and MED 1 (Figure 6A). Protein quantification confirmed that TAF 1, TBP, and MED 1 were less abundant in TAP-n208 samples compared to TAP-wtICP4 samples (Figure 6B). 

### Novel ICP4-Containing Complexes

In addition to TFIID and Mediator, other transcription complexes were isolated with TAP-wtICP4. These include TFIIH, the cleavage and polyadenylation specificity factor (CPSF) complex, and chromatin remodeling complexes. These factors have roles in initiation, elongation, and mRNA processing, implying that ICP4 may function at stages of transcription beyond initiation.

TFIIH is a multi-subunit protein involved in promoter clearance [32-35]. It is composed of 5 subunits that form a core, and a dissociable catalytic activating kinase (CAK) module composed of MAT1, cyclin H, and CDK7. All 5 of the core components of TFIIH were identified by LC-MS/MS in TAP-wtICP4 samples at 6 hpi, while cyclin H and CDK7 of the CAK were not detected (Table 2). The kinase domain is partially responsible for phosphorylating the C-terminal domain of RNA PolII. Its absence in TAP-wtICP4 samples may reflect the observation that RNA PolII is alternatively phosphorylated in HSV infections [36]. Western blot analyses using antibodies against p80 (XPD) and p62 from the core of TFIIH confirmed their presence in the TAP-wtICP4 samples (Figure 6A). As Mediator is involved in recruiting TFIIH to preinitiation complexes [37], it is possible that TFIIH is present in the ICP4-containing complexes because it interacts with Mediator. 

**Table 2:** Novel complexes copurifying with ICP4 (spectral counts).

			3hpi		6hpi				12hpi	
		M.W.	KOS	TAP-ICP4	KOS	TAP-ICP4	n208	TAP-n208	KOS	TAP-ICP4
TFIIH	XPB	89	14	18	8	23	5	5	0	16
	XPD	87	0	11	0	13	2	0	0	2
	p62	62	2	4	2	12	2	12	0	7
	p52	44	7	9	3	11	0	11	0	9
	p44	44	5	7	0	16	0	4	0	6
	MAT1	36	0	0	0	5	0	6	0	3
polyA	CPSF1	166	9	20	5	18	22	51	5	31
	CPSF2	88	6	16	0	12	9	0	3	31
	CPSF3L	30	0	11	4	8	6	3	2	10
	CPSF4	30	2	9	0	8	0	16	0	7
	CPSF7	52	4	6	0	8	7	10	0	6
	FIP1L1	67	0	0	0	8	9	7	9	20
SWI/SNF	Brahma	146	0	0	0	8	0	4	0	0
	SMARCA4	184	0	3	0	9	0	4	0	4
	SMARCC2	107	0	0	0	0	0	6	0	5
	BAZ1B kinase	171	2	16	0	0	0	5	0	14
NURD	CHD3	222	0	18	0	12	0	7	0	34
	CHD4	198	0	13	0	0	0	4	0	0
	HDAC2	55	0	7	0	4	0	4	0	0
Ino80	RUVBL1	50	4	6	0	23	8	12	0	24
	RUVBL2	51	0	6	2	25	5	11	0	25
	ARP5	68	0	0	0	5	0	3	0	10

M.W. Molecular weight predicted from the primary amino acid sequence.

**Figure 7:**
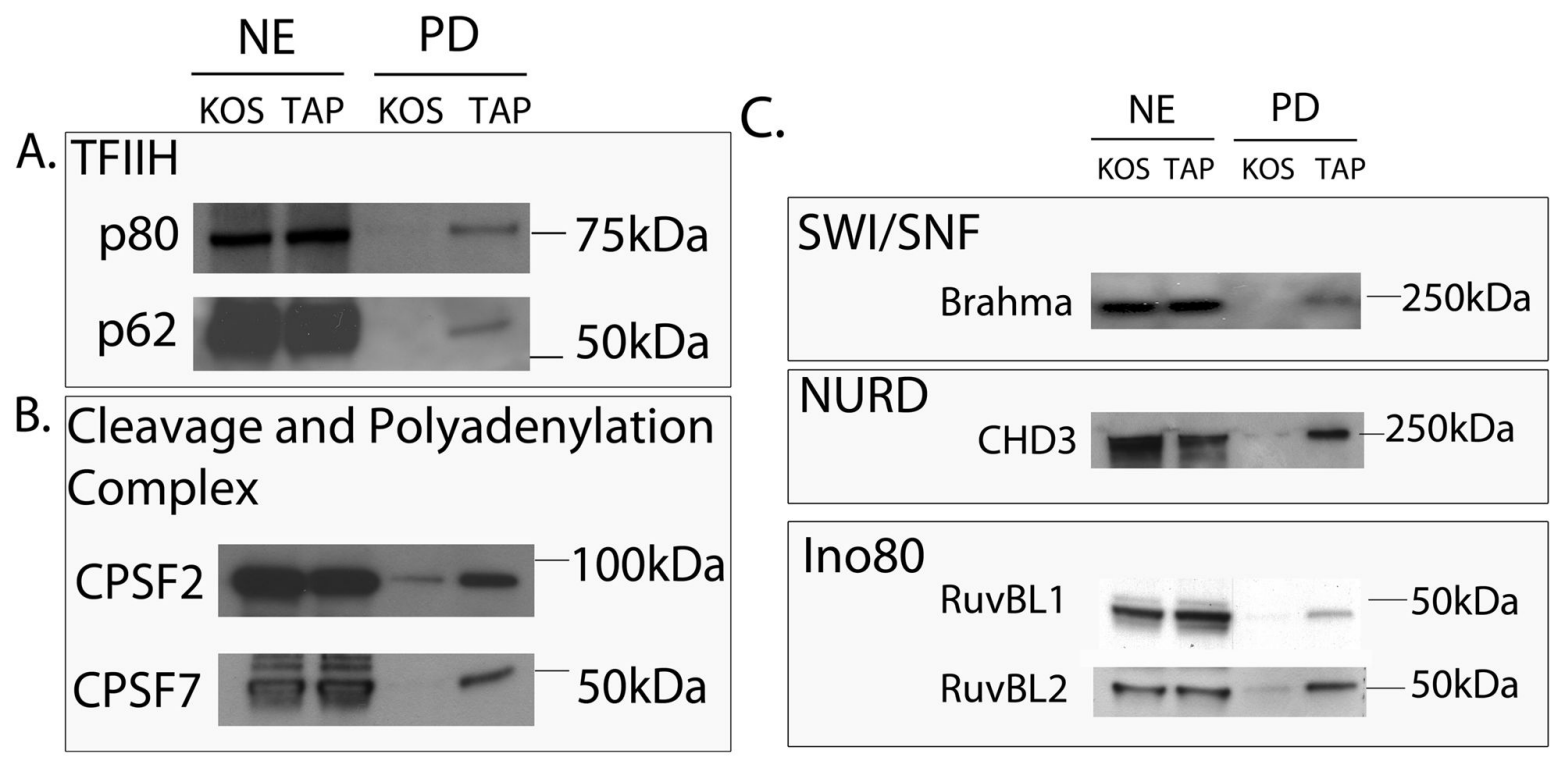
ICP4 containing complexes include TFIIH, cleavage and polyadenylation complex and chromatin remodeling complexes. Western blot analysis of nuclear extracts (NE) or affinity purified (PD) samples collected at 6 hpi using either wtICP4 (KOS) or TAP-wtICP4 (TAP) expressing viruses. Antibodies directed against (A) p80 and p62 of TFIIH, (B) CPSF2 and CPSF7 of the cleavage and polyadenylation complex and (C) Brahma of Swi/Snf, CHD3 of the Nurd complex, and RuvBL1 and RuvBL2 of the Ino80 complex were used.

Components of the cleavage and polyadenylation specificity factor complex (CPSF) were also isolated in TAP-wtICP4 samples (Table 2). The CPSF complex is responsible for recognizing and cleaving the pre-mRNA such that the PolyA polymerase can polyadenylate the nascent transcript [38]. All but two of the CPSFs, 5 and 6, were identified by LC-MS/MS. Western blot analyses using antibodies against CPSF2 and CPSF7 confirmed their presence in TAP-wtICP4 samples (Figure 7B). In TAP-n208 samples, components of the CPSF complex were isolated, although again the association did not appear to be as strong as when the C-terminus of ICP4 was present (Table 2). 

In addition to the many components involved in transcription initiation and elongation, chromatin remodeling factors, including the SWI/SNF, Nurd, and Ino80 chromatin remodeling complexes were isolated in TAP-wtICP4 samples (Table 2). Chromatin remodeling complexes hydrolyze ATP to provide the energy to disrupt histone/DNA interactions, thus allowing for histone displacement during transcription, DNA replication, and DNA repair [39]. As such, a common characteristic of chromatin remodeling complexes is the presence of an ATPase domain. The ATPase containing proteins of the SWI/SNF, Nurd, and Ino80 complexes are Brahma related gene 1 (Brg1) & Brahma (Brm), CHD 3 and 4, and RuvBL 1 and 2, respectively [39]. With the exception of Brg1, each of these ATPases co-purified with TAP-wtICP4 (Table 2). The isolation of these ATPases was confirmed by Western blot analysis with antibodies directed against Brm, CHD 3, RuvBL 1 and RuvBL 2 (Figure 7C). In addition to the ATPase containing proteins, multiple adaptor proteins from each of these complexes were isolated, including SMARCA4, SMARCC2, and Bzb1 kinase from the SWI/SNF complex, HDAC2 from the Nurd complex, and Arp5 from the Ino80 complex (Table 2). 

## Discussion

 The studies presented herein aimed to isolate transcription complexes associated with ICP4 throughout infection to provide a better understanding of the mechanisms underlying expression of the HSV-1 genome. The data demonstrated that i) ICP4 copurified with TFIID complexes containing TBP and 12 TAFs, ii) the core TAFs of TFIID were the most abundant copurifying with wtICP4, iii) components from each module of Mediator, including the kinase module, were isolated with ICP4 while Med 26 was not, iv) ICP4-Mediator complexes formed later in infection relative to ICP4-TFIID complexes, v the C-terminus of ICP4 was not strictly required for interactions with any of the transcription complexes identified, and vi) factors involved in chromatin remodeling, initiation, elongation, and mRNA processing were isolated with ICP4. Together these data suggest a more integrated role for ICP4 in co-transcriptional events during viral infection and provide testable models by which ICP4 may regulate gene expression. 

### TAFs associated with ICP4

 We have previously shown that ICP4 interacts directly with TBP and TAF1 in vitro [17,18]. Affinity purification of ICP4 from HSV infected cells resulted in the isolation of TBP and TAFs1, 2, 3, 4, 5, 6, 7, 8, 9B, 10, 11, and 12. The numbers of spectra counts of ICP4 associated TAFs were among the greatest of all the protein identified in this study, suggesting that TFIID is the major interacting partner of ICP4. Additionally, the abundances of the core TAFs, 4, 5, 6, 9B, and 12 were greater than those of the non-core TAFs in these samples. It has been proposed that the core TAFs are present twice within holo-TFIID [40,41]; therefore, the abundance of the core TAFs in TAP-wtICP4 samples may simply reflect the stoichiometry of the isolated TFIID. An alternative hypothesis is that the abundance of core TAFs is the consequence of a direct interaction between ICP4 and one or more of the core components of TFIID. Interestingly, only TAF4 and TAF9B were abundant in both TAP-wtICP4 and TAP-n208 samples. TAF9B is distinct from TAF9, but like TAF9 forms a histone like pair with TAF6 and is involved the transcription of a distinct but overlapping set of cellular genes relative to TAF9 [42]. Likewise, TAF4 and TAF4B are distinct, form a histone like pairs with TAF12 and are involved in TFIID binding to a distinct set of cellular genes [43]. The precise definition of the common characteristics of cellular genes that are differentially affected by TAFs4 and 9B are not known at present, but it is possible that they might share characteristics with viral or cellular gene that ICP4 regulates. The selection of TAF4 and TAF9B in ICP4 containing complexes also suggests a possible direct interaction between ICP4 and these TAFs. Experiments to test this hypothesis are ongoing.

## Functions of Mediator in HSV infection

 Mediator is present in at least two distinct forms in cells; one that is in complex with the kinase module and is generally associated with repression, and another, which lacks the kinase module, contains Med 26, associates with PolII, and is generally considered a coactivator [29]. The regulatory functions of the kinase module are currently unclear. Many studies implicate the kinase module as being repressive in nature, citing phosphorylation patterns of RNA PolII or cyclin H of TFIIH [44], steric inhibition between Mediator and RNA PolII [30,45,46], or prevention of subsequent rounds of initiation from a re-initiation transcription scaffold [31] as causes. Conversely, other studies suggest a role for the kinase module in the activation of transcription. These studies have demonstrated the presence of the kinase module on active gene promoters [47], and, in direct contrast to other studies, the importance of the kinase module in subsequent rounds of transcription from re-initiation scaffolds [48,49]. 

In the studies presented herein, the Mediator components isolated with wtICP4 were distributed across the head, middle, tail, and kinase modules, with all four subunits of the kinase module identified (Table 1). Furthermore, Med 26 was not detected in any of the independent preparations (Figure 3 and Table 1). Additionally, the majority of the components of Mediator were not isolated until 6 hpi, well after E gene expression and during the onset of late gene expression (Table 1). In light of the studies implicating a repressive function for the kinase module in gene expression, these data may suggest that Mediator is involved in the repression of HSV IE and E gene transcription. During the transition from E to L transcription, a trigger, such as a structural change, posttranslational modification, or an E gene product, may cause ICP4 to recruit or stabilize the kinase domain of Mediator to E promoters, thus resulting in both a direct association between ICP4 and Mediator and the repression of E genes later in infection. Taatjes and colleagues have suggested that the kinase module acts as a molecular switch on highly expressed gene promoters to reduce transcription levels when necessary [31]. Accordingly, ICP4-Mediator interactions would act as a molecular switch to attenuate IE and E transcription. 

Much of the supposition for Mediator functioning in a repressive manner is based on the presence of the kinase module and the absence of Med 26 in TAP-wtICP4 samples. Affinity purification of other activators such as VP16 and SREBP-1 yield both Med 26 and all of the components of the kinase module [50]. Thus, while a repressive function for Mediator in HSV gene expression is intriguing, an activator function for the kinase module cannot be discounted. As mentioned previously, an activation function for the kinase module through the formation of a re-initiation scaffold was recently demonstrated [49]. Furthermore, through interactions between the reinitiation scaffold [51] and the CPSF complex, a ‘gene loop’ can be formed, which brings terminator and promoter in close proximity, resulting in faster reinitiation kinetics [52,53]. TFIID, TFIIH, Mediator, and the CPSF complex, four of the seven components comprising the gene loop, were isolated in TAP-wtICP4 samples. Furthermore, PC4, a cellular factor that shares significant homology to the N-terminus of ICP4 [54] mediates Pol III directed transcription by the formation of gene loops [55], suggesting that ICP4 could be part of a re-initiation scaffold or a gene loop. 

The association of the chromatin remodeling complexes with ICP4 was more prevalent late in infection. Ino80 components are found to higher extents later in infection (6 and 12 hpi) when viral DNA is replicating, and not in cases where DNA replication is less abundant such as early in infection or in n208 infected cells (Table 2). Since the Ino80 complex has been implicated in stabilizing replication forks during DNA replication [56,57], the recruitment of Ino80 and possibly other chromatin remodeling complexes by ICP4 may facilitate DNA replication and late gene expression. 

## The C-terminus of ICP4 Stabilizes Transcription Complexes

 The ICP4 C-terminal mutant, n208, displays a marked reduction in L gene expression and a 100-fold reduction in virus production [23]. We hypothesized that that in the absence of the C-terminus, ICP4 would be defective for interactions required for L gene expression. However, many of the same transcription complexes, including TFIID, Mediator, TFIIH, CPSFs, and chromatin remodeling complexes were isolated with TAP-wtICP4 and TAP-n208, albeit many of the constituents of these complexes were less abundant in the TAP-n208 samples. Based on these observations we conclude that the N-terminal 774 amino acids of ICP4 are sufficient for the formation of all of the transcription complexes that were identified in this study. These results are consistent with previously published data from our lab suggesting that the N-terminus of ICP4 is required for interactions with the transcription machinery, and that the C-terminus augments the functions of the N-terminus, partially by stabilizing these interactions [26,58]. Thus, the severe reduction in L gene expression observed in n208-infected cells may be the result of the inability of the molecule to efficiently stabilize TFIID or Mediator interactions. Unlike wtICP4, n208 is unable to oligimerize on DNA, thus reducing its affinity for DNA [59]. This may result in the reduced formation of ICP4-containing transcription complexes on viral promoters, particularly after viral DNA replication. 

The affinity purification of numerous specific components involved in gene transcription, but the absence of certain notable proteins, such as components of RNA polII, TFII A, B, E, and F, other strong DNA binding proteins, and viral proteins, supports that the majority of the proteins identified are *bona fide* components of ICP4-containing complexes present in infected cells. The diversity and number of components involved in the initiation, elongation, and processing of transcription that were isolated in ICP4-containing complexes suggests that ICP4 is involved in steps beyond simply the formation of initiation complexes, and plays a more determining role in the cascade of HSV gene expression. 
